# A hybrid multi objective cellular spotted hyena optimizer for wellbore trajectory optimization

**DOI:** 10.1371/journal.pone.0261427

**Published:** 2022-01-27

**Authors:** Kallol Biswas, Amril Nazir, Md. Tauhidur Rahman, Mayeen Uddin Khandaker, Abubakr M. Idris, Jahedul Islam, Md. Ashikur Rahman, Abdul-Halim M. Jallad

**Affiliations:** 1 Department of Electrical and Electronic Engineering, Universiti Teknologi PETRONAS, Tronoh, Perak, Malaysia; 2 Department of Information Systems and Technology Management, College of Technological Innovation, Abu Dhabi Campus, Zayed University, Abu Dhabi, United Arab Emirates; 3 Department of Petroleum Engineering, Universiti Teknologi PETRONAS, Tronoh, Perak, Malaysia; 4 Centre for Applied Physics and Radiation Technologies, School of Engineering and Technology, Sunway University, Bandar Sunway, Selangor, Malaysia; 5 Department of Chemistry, College of Science, King Khalid University, Abha, Saudi Arabia; 6 Research Center for Advanced Materials Science (RCAMS), King Khalid University, Abha, Saudi Arabia; 7 Department of Fundamental and Applied Sciences, Universiti Teknologi PETRONAS, Tronoh, Perak; 8 Department of Electrical Engineering, United Arab Emirates University, Al Ain, United Arab Emirates; 9 National Space Science and Technology Center, United Arab Emirates University, Al Ain, United Arab Emirates; Universiti Sains Malaysia, MALAYSIA

## Abstract

Cost and safety are critical factors in the oil and gas industry for optimizing wellbore trajectory, which is a constrained and nonlinear optimization problem. In this work, the wellbore trajectory is optimized using the true measured depth, well profile energy, and torque. Numerous metaheuristic algorithms were employed to optimize these objectives by tuning 17 constrained variables, with notable drawbacks including decreased exploitation/exploration capability, local optima trapping, non-uniform distribution of non-dominated solutions, and inability to track isolated minima. The purpose of this work is to propose a modified multi-objective cellular spotted hyena algorithm (MOCSHOPSO) for optimizing true measured depth, well profile energy, and torque. To overcome the aforementioned difficulties, the modification incorporates cellular automata (CA) and particle swarm optimization (PSO). By adding CA, the SHO’s exploration phase is enhanced, and the SHO’s hunting mechanisms are modified with PSO’s velocity update property. Several geophysical and operational constraints have been utilized during trajectory optimization and data has been collected from the Gulf of Suez oil field. The proposed algorithm was compared with the standard methods (MOCPSO, MOSHO, MOCGWO) and observed significant improvements in terms of better distribution of non-dominated solutions, better-searching capability, a minimum number of isolated minima, and better Pareto optimal front. These significant improvements were validated by analysing the algorithms in terms of some statistical analysis, such as IGD, MS, SP, and ER. The proposed algorithm has obtained the lowest values in IGD, SP and ER, on the other side highest values in MS. Finally, an adaptive neighbourhood mechanism has been proposed which showed better performance than the fixed neighbourhood topology such as L5, L9, C9, C13, C21, and C25. Hopefully, this newly proposed modified algorithm will pave the way for better wellbore trajectory optimization.

## Introduction

The increasing demand for energy consumption around the world has promoted the depletion of conventional energy sources. Hence, the high energy demand has drawn attention to producing energy from unconventional sources. However, producing oil/gas from unconventional sources is not easier with conventional production methods. Now, with the advancement of technologies, such as directional/horizontal drilling, the production from unconventional sources has become possible with some unwanted challenges [1]. One of the major challenges in directional / horizontal drilling is wellbore trajectory design, which is associated with cost and safety [2]. Sometimes, directional/horizontal drilling needs high expenditure, which tends to increase the oil and gas price at the consumer level. Therefore, to minimize the oil/gas price, it is crucial to minimize the operational expenditure. In directional/horizontal drilling, one of the key ways to reducing operational expenditure is optimizing the wellbore trajectory [3].

Wellbore trajectory is the direction in which the wellbore is drilled. To reach a sub-surface target, there can be thousands of probable well paths. However, the success of directional drilling depends on choosing the best path, which can be only done by the optimization of wellbore trajectory. A wellbore trajectory can be optimized considering several parameters; among them, length, torque, energy, rate of penetration, separation factor is the most influential [4–9]. Some pieces of research optimized the wellbore trajectory by considering one parameter [7]. However, this single objective optimization could not be able to provide enough cost efficiency and safety to the wellbore. Therefore, to increase cost efficiency and safety, multi-objective optimization was introduced by several researchers [5, 10–13]. In multi-objective optimization, two or more parameters were considered for wellbore trajectory optimization. Each of these parameters is optimized considering several tuning variables such as azimuth angle, dogleg severity, inclination angle, and kick-off point.

To optimize the above-mentioned parameters several algorithms were utilized with some drawbacks such as less exploitation capability, local optima trapping, nonuniformed distribution of non-dominated solutions, and disability of tracking isolated minima [11]. The researchers focused on metaheuristic algorithms due to the weakness of the traditional algorithms in the large search region [14, 15]. Among the metaheuristic algorithms, genetic algorithms (GA), particle swarm optimization (PSO), ant colony optimization (ACO), artificial bee colony optimization (ABC), harmony search (HS) were utilized for well trajectory optimization [2, 4, 16]. To improve the issues faced by the metaheuristic algorithms and to improve the efficiency, some hybrid algorithms, for example, hybrid cuckoo search optimization (HCSO), hybrid bat flight optimization (HBFO) were introduced [17–19]. Due to the hybridization, these algorithms showed some significant improvements in the exploration capabilities [11]. However, these improvements enabled the algorithms to provide better solutions but on the other side, those make the convergence speed slower. Therefore, still, improvement is indispensable for increasing the exploitation capabilities of these algorithms.

In recent times, multi-objective genetic algorithm (MOGA), multi-objective cellular particle swarm optimization (MOCPSO), multi-objective cellular grey wolf optimization and particle swarm optimization (MOCGWOPSO) have been used for length, torque, and well-profile energy optimization [5, 10, 11]. However, MOGA and MOCPSO have faced exploitation related problems. On the other side, MOCGWOPSO provided excellent non-dominated solutions for length and torque, but it showed weakness in the case of well-profile energy optimization during multi-objective optimization. From the above discussion, it can be concluded that hybridization of any standard algorithms makes the algorithms more effective and efficient for wellbore trajectory optimization. Hence, this research focuses on the hybridization of the spotted hyena optimization (SHO) algorithm for wellbore trajectory optimization.

SHO is a newly designed metaheuristic algorithm that is inspired by the hunting mechanism of the spotted hyenas [20, 21]. This algorithm has a very high convergence rate. Therefore, this algorithm faced local optima trapping issue during non-linear optimization. To overcome this issue herein cellular automata (CA) has been incorporated with SHO in this work [22]. CA is incorporated due to its slow diffusion mechanism and information exchanging capability among the neighbours. The slow diffusion mechanism helps to avoid the local optima trapping issue and the information exchanging capabilities enhance the local search capability of SHO. Besides, the velocity update mechanism of PSO has been incorporated to enhance the hunting capability of SHO. Moreover, an adaptive neighbourhood mechanism has been proposed and compared with the fixed neighbourhood topology structure such as L5, L9, C9, C13, C21, and C25 in this work. The performance analysis of the proposed algorithms for wellbore trajectory optimization has been done by comparing with the previously used MOCPSO and other states of the art algorithms such as MOSHO, MOCGWO [21, 23]. This comparative analysis has been conducted based on some statistical analysis. The proposed algorithm has achieved the lowest value of IGD, SP, ER. This indicates that the obtained Pareto front by the proposed algorithm is nearer to the true Pareto front, the non-dominated solutions are nearly spaced, and it has a very less number of isolated minima. The Spearman correlation coefficient test has also been performed to analyze the sensitivity of each decision variable on the three objectives [24]. This proposed algorithm will pave the way to design a less complex and cost-effective wellbore trajectory.

## Mathematical formulation

Up to the date, several methods (radius of curvature method (RCM), tangential method, angle averaging method, minimum curvature method) were utilized for characterizing the directional well design parameters [25, 26]. In this work, the RCM method is used to formulate the three-objective functions [25, 27].

To compute the trajectory length, radius curvature method was utilized considering several parameters such as azimuth angle, hold angle, vertical inclination, lateral length, true vertical depth, and dogleg severity. The following formulas are used to compute the constant of curvature and radius of curvature between two points in RCM.


$$a=\frac{1}{\Delta M}\sqrt{({\theta_{2}-\theta_{1})}^{2}{sin}^{4}(\frac{\left(\emptyset_{2}+\emptyset_{1}\right)}{2})+{\left(\emptyset_{2}+\emptyset_{1}\right)}^{2}}$$ (1)

$$r=\frac{1}{a}=\frac{180*100}{\pi *T}$$ (2)

$$\Delta M=r*\sqrt{\left(({\theta_{1}-\theta_{2})}^{2}{sin}^{4}(\frac{\left(\emptyset_{1}-\emptyset_{2}\right)}{2})+{\left(\emptyset_{1}-\emptyset_{2}\right)}^{2}\right)}$$ (3)

**Fig 1:**
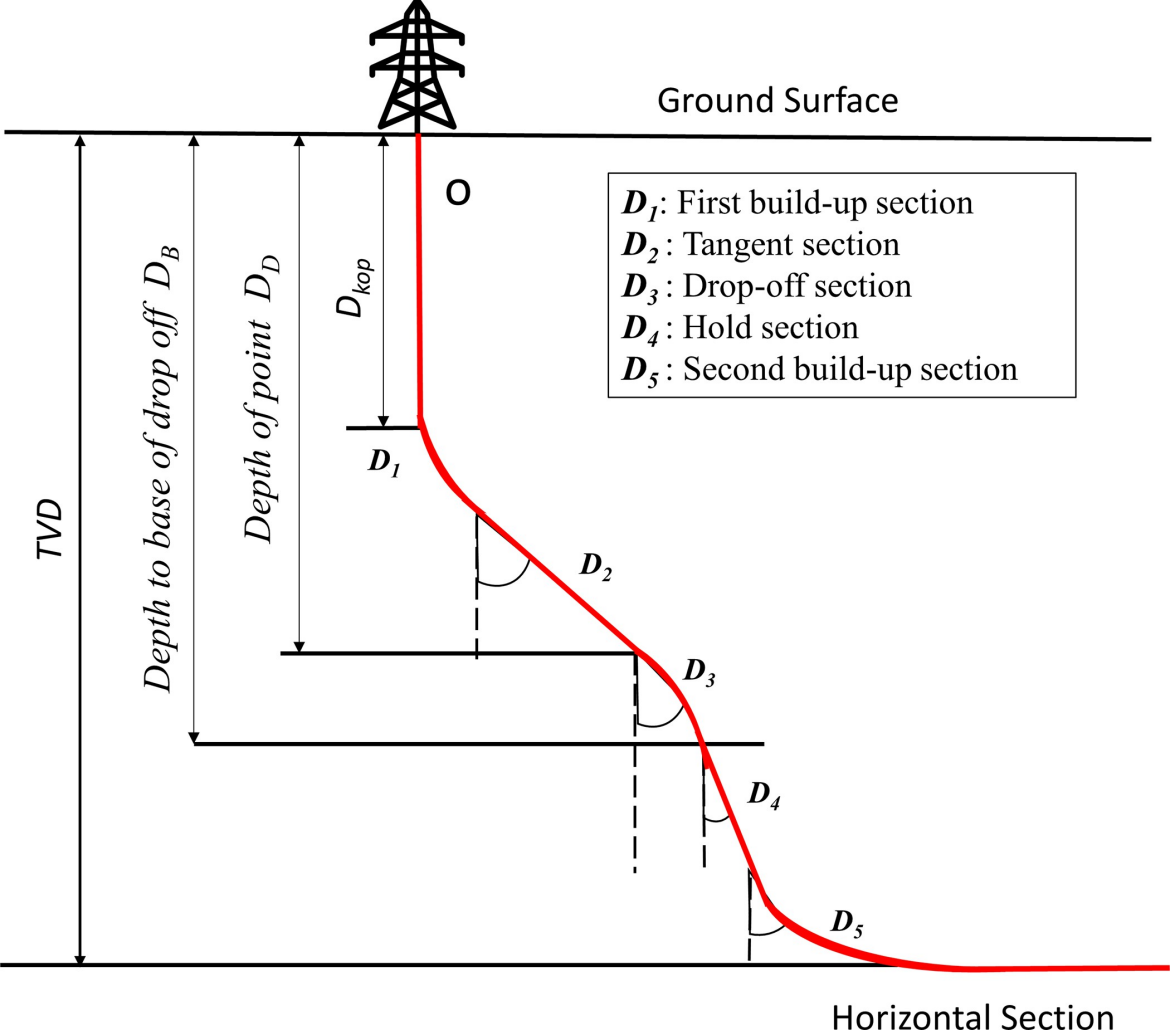
An overview of the wellbore trajectory.

Herein, a and r represents the constant of curvature and radius of curvature, respectively. The dogleg severity, inclination angle, and azimuth angle are denoted by *T*, ∅_*i*_, and *θ*_*i*_, respectively. Δ*M* is the 3D well path between two points. The general vertical plane for a wellbore trajectory with various straight and curved sections is depicted in Fig 1.

According to Fig 1, the TMD’s fundamental equation is composed of seven segments which are represented by Eq (4).


$$TMD=D_{kop}+D_{1}+D_{2}+D_{3}+D_{4}+D_{5}+HD$$ (4)

Where *D*_*kop*_ represents the estimated length of kick-of section, build and drop segments are represented by (*D*_1_, *D*_3_ *and D*_5_), tangent segment, hold segment and horizontal section are represented by (*D*_2_), (*D*_4_) and HD respectively. The torque and well-profile energy can be characterized by the following equations.


$$Torque=T_{vertical}+T_{1}+T_{2}+T_{3}+T_{4}+T_{5}+T_{6}+T_{7}$$ (5)

$$E=D_{1}\emptyset_{1}^{2}+D_{3}{\left(\emptyset_{2}-\emptyset_{1}\right)}^{2}+D_{5}{\left(\emptyset_{3}-\emptyset_{2}\right)}^{2}$$ (6)

In this work buoyancy factor *B* = 0.7, weight of unit pipe length *w* = 0.3*KN*/*ft*,friction factor *u* = 0.2 and pipe diameter *D* = 0.2*ft* have been used for the calculation. The detail description of mathematical formulation was demonstrated in our previous work [11].

### Constraints

The optimization of the wellbore trajectory is constrained in two ways: by operational constraints and by the upper and lower limits of 17 tuning variables. The tuning variables’ upper and lower limits are listed in Table 1. However, in this work the operational constraints are the true vertical depth (TVD), and the casing setting depth, C1, C2, C3. During the trajectory optimization, the following equation should also be satisfied.

**Table 1:** Operational and variable’s constraints for wellbore trajectory design.

Variables	Variable constraints imposed on Wellbore design
Target True Vertical Depth (TVD)	Min. = 10850ft. and Max. = 10950ft.
Lateral Section length (LSL) HD.	2500ft.
Dogleg Severity	$T_{1}\leq \frac{5^{0}}{100\;ft.};\;T_{2}\leq \frac{5^{0}}{100\;ft.};\;T_{3}\leq \frac{5^{0}}{100\;ft.};\;T_{4}\leq \frac{5^{0}}{100\;ft.};\;T_{5}\leq \frac{5^{0}}{100\;ft.}$
Minimum value of inclination angles	∅_1_ = 10^0^; ∅_2_ = 40^0^; ∅_3_ = 90^0^
Maximum value of inclination angles	∅_1_ = 20^0^; ∅_2_ = 70^0^; ∅_3_ = 95^0^
Minimum value of azimuth angles	*θ*_1_ = 270^0^; *θ*_2_ = 270^0^; *θ*_3_ = 270^0^; *θ*_4_ = 330^0^; *θ*_5_ = 330^0^; *θ*_6_ = 355^0^
Maximum value of azimuth angles	*θ*_1_ = 280^0^; *θ*_2_ = 280^0^; *θ*_3_ = 280^0^; *θ*_4_ = 340^0^; *θ*_5_ = 340^0^; *θ*_6_ = 360^0^
Kick off point depth (TVD)	Min. *D*_*kop*_ = 600*ft*.; Max. *D*_*kop*_ = 1000*ft*.
Second build point depth (TVD)	Min. *D*_*D*_ = 6000*ft*.; Max. *D*_*D*_ = 7000*ft*.
Third build point depth (TVD)	Min. *D*_*B*_ = 10000*ft*.; Max. *D*_*B*_ = 10200*ft*.
Casing setting depth after first build	Min. *C*_1_ = 1800*ft*.; Max. *C*_1_ = 2200*ft*.
Casing setting depth after second build	Min. *C*_2_ = 200*ft*.; Max. *C*_2_ = 8700*ft*.
Casing setting depth after third build	Min. *C*_3_ = 10300*ft*.; Max. *C*_3_ = 11000*ft*.


$$TVD=Y_{1}+Y_{2}+Y_{3}+Y_{4}+Y_{5}+Y_{6}$$ (7)

Herein, the vertical depth of each subsection at each drop off point is denoted by the symbol *Y*_*i*∈(1,6)_. There are also some non-negative constraints [10].

**Fig 2:**
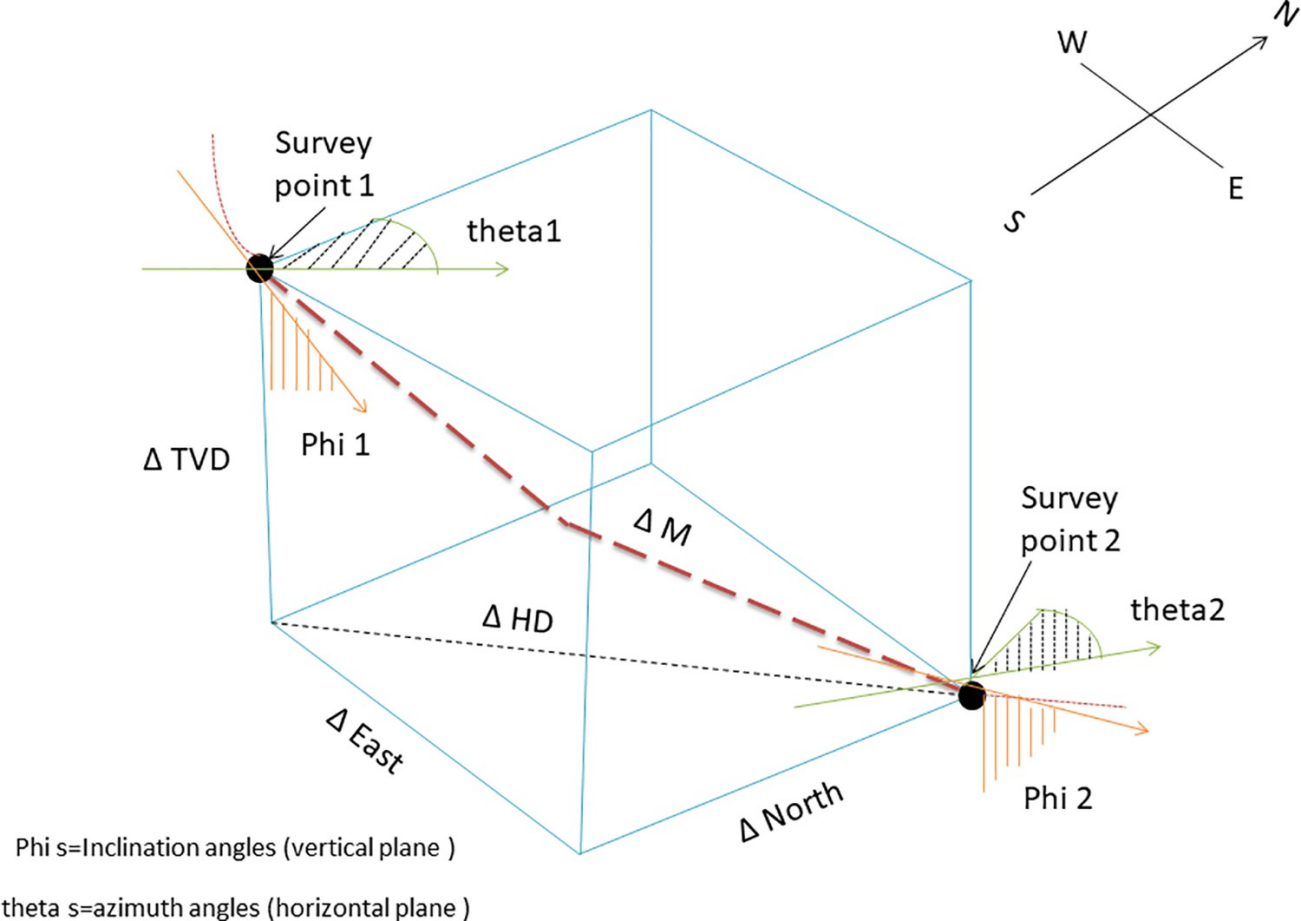
Offset distance of a typical directional wellbore trajectory.


Fig 2 illustrates the trajectory’s deviated direction to the east, north, and vertical sides. The offset distance in these three directions is derived following the radius of curvature method.

$$\Delta North=\frac{\Delta M(\cos\left(\emptyset_{1}\right)-\cos\left(\emptyset_{2}\right)).(\sin\left(\theta_{2}\right)-\sin\left(\theta_{1}\right))}{{(\emptyset }_{2}-\emptyset_{1}).(\theta_{2}-\theta_{1})}$$ (8)

$$\Delta East=\frac{\Delta M(\cos\left(\emptyset_{1}\right)-\cos\left(\emptyset_{2}\right)).(\cos\left(\theta_{1}\right)-\cos\left(\theta_{2}\right))}{{(\emptyset }_{2}-\emptyset_{1}).(\theta_{2}-\theta_{1})}$$ (9)

$$\Delta Vertical=\frac{\Delta M(\sin\left(\theta_{2}\right)-\sin\left(\theta_{1}\right))}{{(\emptyset }_{2}-\emptyset_{1})}$$ (10)

The above mentioned three Eqs (8–10), calculates the distance of east-west, north-south, and TVD. Another important constraint is the casing setting depth which has a direct impact on cost. Therefore, it is necessary to consider the following constraints during trajectory optimization.


$${TVD}_{min}<TVD<{TVD}_{max}$$

$$C_{1,min}<C_{1}<C_{1,max}$$

$$C_{2,min}<C_{2}<C_{2,max}$$

$$C_{3,min}<C_{3}<C_{3,max}$$

## Framework for wellbore trajectory optimization

In this multi-objective optimization, the proposed hybrid algorithm needs to optimize length, torque, and well profile energy from Eqs (4–6). The algorithm needs to fine-tune all the 17 tuning variables for optimizing these three objectives parallelly. Table A1 in S1 Appendix tabulated the bounds and explanation of these variables. The overall multi-objective optimization process can be mathematically expressed as follows.


$$arg\;\underset{i\in (1,2,3)}{\min}f_{i}(X);\;X\in \{x_{j}\}\;where\;j=1,2,\ldots 17$$ (11)

In the proposed algorithm diffusion mechanism of CA will be used to increase the exploration capability of SHO, later velocity update equation of PSO will be used. This will improve the hunting mechanism of SHO.

### Spotted hyena optimizer

The mechanism of SHO will be discussed in this section. It is a recently proposed metaheuristic algorithm that was inspired by the hyena’s prey hunting mechanism [20, 21]. Usually, the SHO algorithm imitates the behaviours of a consistent hyena cluster. It consists of four main steps such as searching, encircling, hunting and attacking. The group of fellow peers is directing the hunting behaviour toward the best solution, and preserve the best results. The following equations are used to replicate the encircling interactions of spotted hyenas.


$$\vec{M_{h}}=|\vec{A}.\vec{Q_{p}}(x)-\vec{Q}(x)|$$ (12)

$$\vec{Q}(x+1)=\vec{Q_{p}}(x)-\vec{F}.\vec{M_{h}}$$ (13)

Where $\vec{M_{h}}$ define the distance to the prey from the spotted hyena, *x* represents the present iteration, $\vec{A}$ and $\vec{F}$ are the coefficient vectors, $\vec{Q_{p}}$ specifies the prey’s position vector, $\vec{Q}$ indicates the hyena’s position vector. Besides || and “.” respectively represent the absolute value and vector multiplication. The following formulas are used to calculate the vector $\vec{A}$ and $\vec{F}$.


$$\vec{A}=2.r\vec{d_{1}}$$ (14)

$$\vec{F}=2\vec{h}.r\vec{d_{2}}-\vec{h}$$ (15)

$$\vec{h}=5-(Iteration*(\frac{5}{{Max}_{Iteration}})$$ (16)

Where, Iteration = 1,2, 3,.. *Max*_*Iteration*_, In [0,1], vectors $r\vec{d_{1}}$ and $r\vec{d_{2}}$ are random. The search agent can explore different parts of the search space by adjusting the values of vectors $\vec{A}$ and $\vec{F}$. On the other hand a hyena can adjust its position around its prey by applying Eqs (14–16). This algorithm stores the best solutions and compels others to upgrade their positions. When the value becomes $|\vec{F|}<1$ then the algorithm enables the hyenas to attack the target. The following equations are defined to replicate the hunting behaviour of hyenas and to identify the feasible search space regions.


$$\vec{M_{h}}=|\vec{A}.\vec{Q_{h}}-\vec{Q_{k}}|$$ (17)

$$\vec{Q_{k}}=\vec{Q_{h}}‐\vec{F}.\vec{M_{h}}$$ (18)

$$\vec{D_{h}}=\vec{Q_{k}}+\vec{Q_{k+1}}+\ldots \vec{Q_{k+N}}$$ (19)

Where $\vec{Q_{h}}$ denotes the first best-spotted hyena’s position, $\vec{Q_{k}}$ defines the specific location of other search agents. The number of spotted hyenas is represented by N which is calculated as follows:

$$N={count}_{nos}(\vec{Q_{h}},\vec{Q_{h+1}},\vec{Q_{h+2}},\ldots \ldots ..(\vec{Q_{h}}+\vec{G})$$ (20)

Where $\vec{G}$ is a random vector in [0.5,1], nos denotes the number of solutions after $\vec{G}$ is added However in a certain search space these solutions are almost the same as the best optimum solutions. Moreover $\vec{D_{h}}$ is a group of N number of optimal solutions.

#### Exploitation

Like every algorithm SHO also has to perform exploration and exploitation. During exploitation, the value of $\vec{h}$ gradually decreases from 5 to 0. The variations in $\vec{F}$ also contribute to exploitation. The algorithm allows the hyenas to attack the prey when it becomes $|\vec{F|}<1$. The formulation can be expressed as follows:

$$\vec{Q}(x+1)=\frac{\vec{D_{h}}}{N}$$ (21)

Where $\vec{Q}(x+1)$ registers the best position and helps the search agent to update their position according to the best search agent. It permits all of its search agents to attack the prey.

#### Exploration

The hyenas of vector $\vec{D_{h}}$ mostly guide the exploration process. During exploration, the value of $\vec{F}$ become $|\vec{F|}>1$ or $|\vec{F|}<‐1$. It generally controls the hyenas for global search. The value of $\vec{F}$ forces agents to deviate from optimal solutions, hence expanding the scope of exploration and local optima avoidance. Another important contributor is the vector $\vec{A}$ which provides random values to the prey in the range of [0.5,1]. Search agents use Eqs (19–21) during optimization to create a cluster towards the best search agent to upgrade their positions. Meanwhile, during iterations, parameters h and $\vec{F}$ reduce linearly. Finally, when the termination condition is matched, the positions of search agents which create a cluster are considered as the optimal solutions.

#### Archive

In multi-objective optimization, the stock of all Pareto optimal solutions (those that have been obtained so far) is defined as an archive. It is comprised of two major components: an archive controller and a grid system.

#### Archive controller

The primary responsibility of this controller is to determine whether or not to include the solution in the archive. The following are some of the most important points to note about the updating mechanism:

- If any single member of the archive dominates it, the solution cannot be included.
- If a new solution is superior to one or more solutions in the archive, it can be incorporated.
- If the new solution and archive solutions do not have any dominance over each other, the new member should be incorporated in the archive.
- The grid method should be used so that one of the crowded solution sections can remove. This will allow to include a new solution that will enhance Pareto’s optimal diversity.

#### Grid mechanism

Grid is a space in which each particle obtains a unique solution to its objective function. An adaptive grid can be considered of as a hypercubic space with a uniform distribution of elements. If the inserted individual is outside the grid’s current bounds, the grid must be reconfigured. The grid mechanism divides the search space into multiple sub-search zones.

#### Group selection mechanism

The challenge of comparing the solutions with archive members in a multi-objective search area is very complex. This process has been performed in this study through the use of a group selection mechanism. The mechanism determines the least populated area inside the solution region. Later in the chapter, it suggests one of the non-dominated solutions from the least populated region to the closest neighbour region. The roulette-wheel method was employed to assist in determining the least-populated area [28]. This method can be defined as

$$U_{k}=\frac{e}{S_{n}}$$ (22)

where *e* stands for a constant number (*e*>1), the total number of Pareto optimal solutions achieved from the *n*_*th*_ segment till now is represented by *S*, n is the number of segments This is a proportionate selection strategy. According to this method, every individual’s fitness value is almost correlated to the range offered by the roulette wheel proportion. $\vec{G}$ is the deciding vector in MOSHO, and it is directly proportional to the number of optimal solution selections. The archive protects the obtained non-dominated solutions against degradation. The conventional algorithm, which is based on the archive, employs a variety of operators (mutation, crossover), which compelled the algorithm to focus more on the archive member. As a result, the variables in MOSHO exchange information with the various solutions in the search space. This enhances the capability for exploration but reduces the capability for convergence. That is why a group selection mechanism has been used to select a minimum of one member from the solution space.

#### Cellular automata

The concept of cellular automata has been described in this section. Von Neumann and Ulam first published the concept of CA [11, 22]. It can be characterized as a distribution of cells excreted in a particular topological structure. They are the cell, the cell state, the cell space, the neighbourhood, and the transition rule [29]. The next status of each cell will be determined by considering the present state of all the neighbouring cells. CA is composed of five components. They are cell, cell state, cell space, neighbourhood, and transition rule [30]. The cell state is a term that refers to the information about the present cell. It assists in determining the next state. Cell space is a collection of cell sets. It has applications in multiple dimensions (one, two, and three). However, because real-world processes are mostly reproduced using finite grids, the cell space boundary must be defined during operation. The limit is a ring grid. That is, the left border will remain connected to the right, and the top border will remain connected to the bottom border. The neighbourhood can be described as a group of cells that surround a centre cell. It is primarily responsible for selecting the following state. The transition rule determines the cell’s next state based on the status of neighbouring cells. The formulation of CA can be characterized as follows.

Usually, an m-dimensional CA can be characterized as a grid of m-dimensional single cells. Each cell has its own value. According to the transition rule, each cell can update its state. Therefore, Q is a cellular automaton that can be formulated as Eq (23).


Q=(T,H,m,g)[WhereQisaquadraple] (23)

Where,

The finite set of state        :T                                                Dimension of Q            :*d* ∈ *Z* +

Neighborhood                :H                    Transition rule            :g

Let’s take i ∈ *Z*^*m*^ is the position of a cell where *m* is the dimension of the latticegrid, then the neighbourhood H can be formulated as

$$H_{i}=(i,\;i+r_{1},\ldots \ldots \ldots ..i+r_{n)}$$ (24)

Where neighbourhood size is represented by n, a fixed vector from the search space of *m-*dimension is represented by *r*_*j*_.

#### Neighbourhood

The term neighbourhood refers to a group of cells that surround a central cell. Additionally, the neighbours can be defined as the other atoms connected by a single atom. The following definitions are provided to illustrate the precise composition of various neighbours. This work defines the grid’s neighbours based on their direction and radius. It is frequently referred to by two structural labels. They are *L*_*n*_ and *C*_*n*_. If there are *n—*1 nearest neighbour around the centre cell which are specific in direction, then the structure can be denoted by *L*_*n*_ or *C*_*n*_.

**Fig 3:**
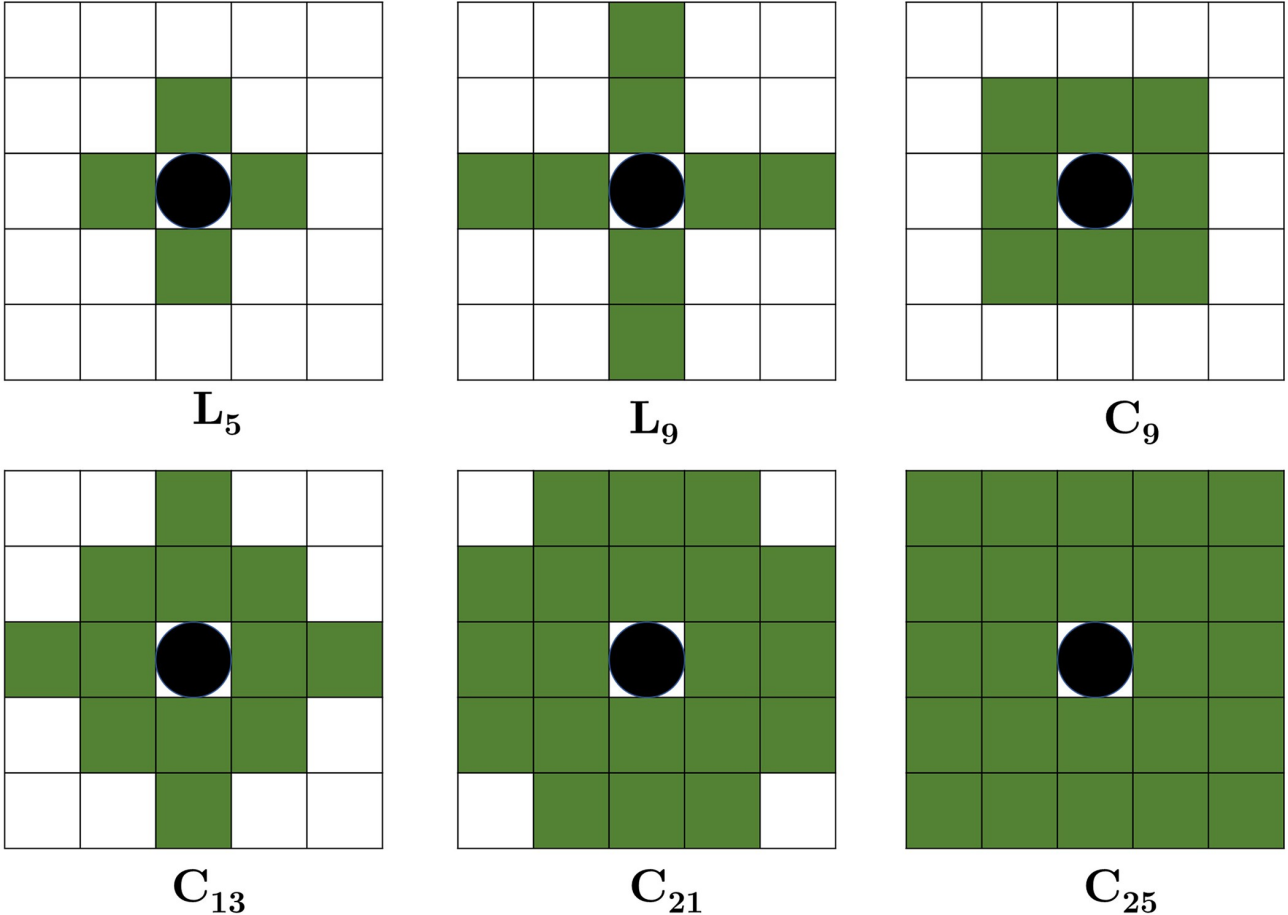
Different types of neighbourhood structure.

If the directions of neighbouring cells are at the top, bottom, left, and right then it will be denoted by *L*_*n*_. If on the other hand, the directions are at the top, bottom, left, right, and diagonal then it will be denoted by *C*_*n*_. Six distinct forms of classical structure were analysed in this study to determine the effect of neighbourhood structure. These structures are represented in Fig 3 as (L5, L9, C9, C13, C21, and C25).

#### Transition rule

It is a set of rules that govern how each atom changes state. The next state of the current atom is decided by evaluating the status of neighbouring atoms. Let us take the best position of the neighbor *M*_*i*_ around an atom as $P_{i}^{t}(M_{n})$. The neighbour with the highest fitness value will be used to facilitate information diffusion and effectiveness. The transition rule can be defined as

$$fitness\;\left(P_{i}^{t+1}(M_{i})\right)=\min\left(fitness\;\left(P_{i}^{t}(M_{i})\right)\ldots .fitness\;\left(P_{i}^{t}(M_{i+r_{1}})\right)\right)$$ (25)

where the present value of *M*_*i*_ is represented by $fitness\;(P_{i}^{t}(M_{i}))$. Therefore neighbour with the best fitness value from the CA structure can be obtained by using Eq (25). Later it will be used to update the state of the centre cell.

### Particle swarm optimization

It is an evolutionary algorithm inspired by the behaviour of a bird’s flock [31]. It offers a number of advantages, including a low mathematical complexity, a high capability for optimization, and easy implementation. PSO is comprised of two distinct processes. Birds carry out both processes. Initially, the bird will conduct a random search for the food source that is the closest to it. Later on, it will utilise its flying experience to determine the location of the meal. The shift in position has been referred to as velocity, and it varies with the passage of time. During the flight, particle speed increased stochastically towards its best point (personal best) and the community’s best solution (global best) [32]. The candidate solution, which is represented by a particle, is a bird. In addition, food acts as a representation of the best possible solution to the problem. In this work *i*^*th*^ particle is considered as *X*_*i*_ = *X*_*i*1_, *X*_*i*2_, *X*_*i*3_….*X*_*id*_, and *v*_*i*_ = *v*_*i*1_, *v*_*i*2_, *v*_*i*3_….*v*_*id*_ represent the velocity of each particle. With their initial velocity each particle start searching in the search space, where *p*_*i*_ = *p*_*i*1_, *p*_*i*2_, *p*_*i*3_….*p*_*id*_ represent the personal best position of each particle and *p*_*g*_ = *p*_*g*1_, *p*_*g*2_, *p*_*g*3_….*p*_*gd*_ represent the global best position of each particle. In PSO each particle updates its velocity and position by using the following equations

$$V_{i}^{t+1}={w\times V}_{i}^{t}+c_{1}.r_{1}(p_{i}^{t}-X_{i}^{t})+c_{2}.r_{2}(p_{g}^{t}-X_{i}^{t})$$ (26)

$$X_{i}^{t+1}=X_{i}^{t}+V_{i}^{t+1}$$ (27)

Here *c*_1_ and *c*_2_ are acceleration coefficient which mostly controls the exploration and exploitation capability of the algorithm, inertia weight is represented by *w*, *r*_1_ *and r*_2_, both represent the random numbers [0,1]. The fitness value is the determinant to analyse the quality of the best particle. The particle which has the best fitness value is taken as the global best solution [33].

### Proposed hybrid algorithm

The framework of the proposed algorithm, as well as the improvement approach, have been discussed in detail in this section.

#### Framework of the MOCSHOPSO algorithm

The purpose of this research is to improve the performance of SHO by utilizing different techniques from CA and PSO for wellbore trajectory optimization problems. One crucial strategy is CA which is utilized to improve the performance of the SHO-PSO. The following three advantages have motivated to implement the hybridization strategy.

- The local search capability can be enhanced with the assistance of CA, as it enhances exploitation capability through interaction with its neighbours. However, the process of information transmission aids in exploration.
- As a result of the candidates’ consequent solutions being attracted to the good SHO solutions, the SHO’s convergence speed is quite fast, resulting in a local optima trap. The slow diffusion mechanism of CA in conjunction with SHO will aid SHO in avoiding the local optima trap.
- The velocity update mechanism of PSO will be utilized to improve the hunting mechanism of SHO. The velocity component of PSO is frequently managed by multiplying the particle’s velocity by a factor. This regulation of velocity is intended to strike a balance between exploration and exploitation.

This programme generates semi-random populations of spotted hyenas. They are distributed in an n-dimensional lattice grid. The operational constraints are managed during the initialization of the agent’s positions in the manner that has been proposed. The wellbore trajectory tuning variables x j are then initialised at random, but in a constrained environment, to get the desired results. If the initial positions satisfy the operational and non-negative constraints, they are accepted without further consideration. The values of the first tuning variable will be less chaotic and semi-randomly created as a result of this method. Then, in terms of wellbore trajectory optimization, the fitness value of each population is computed. Following that, it begins its update loop. It utilises the CA principle to create a new neighbourhood. During the neighbourhood generation process, some neighbours overlap. It enables the algorithm to incorporate an implicit migration mechanism. Additionally, it aids in the seamless diffusion of the best solutions throughout the population. As a result, it can retain a greater degree of diversity than the original SHO. Soft diffusion is critical in maintaining a balance between exploration and exploitation. The entire search space is divided into several sub-search regions by this approach. As a result, they can update the operation separately. However, if the neighbours overlap in this situation, information is transferred on an ad hoc basis.

However, the hunting mechanism of SHO has been modified by using the velocity update mechanism of PSO [34]. Therefore Eq (18) can be expressed as follows.


$$\vec{Q_{k}^{t+1}}=w\times \vec{Q_{k}^{t}}+c_{1}.r_{1}(\vec{Q_{h}}-\vec{M_{h}})+c_{2}.r_{2}(\vec{Q_{h}}-\vec{M_{h}})$$ (28)

The updated hunting mechanism will be used by the proposed algorithm The pseudocode of the proposed algorithm has been expressed as follows.

**MOCSHOPSO**

**Input:** Spotted hyena population,

**Output:** Best search agent

1: Initialize the population of spotted hyena

2: Initialize *h*,*A*,*F*,*N* parameters

3: Evaluate Spotted hyena population

4: Select *Q*_*h*_ = best first search agent

5: Select *D*_*h*_ = Cluster of all obtained solution

6:    w**hile** iteration number < maximum iteration number

7:        **for** i← 1 spotted hyena population

8:            Create neighbors

9:                Update the position of hyena

10:                    Update (h,*A*,*F*,*N*)

11:                        Evaluate spotted hyena’s new position

12:                            **if** new position outperforms

13:                    Replace the current hyena

14:                **end if**

15:            evaluation number ++

16:        **end for**

17:    **end while**

16: **while** iteration<iteration_max_ **do**

17:        **for** each spotted hyena **do**

18:            Position update by using Eq (28)

19:        **end for**

20:    Update (h,*A*,*F*,*N*)parameters

21:        Calculate the fitness value of current spotted hyena

22:    Update *Q*_*h*_ and *D*_*h*_

23:        x = x+1

24: **end while**

**25: return** *Q*_*h*_

#### Adaptive neighbourhood

In the proposed method every search agent will create a neighbourhood according to the fitness value of the search agent. If the search agent has superior quality, then it will create a large size neighbourhood. It will increase the probability of overlapping with the best one. Besides, it will also increase the diversity of the solutions which also helps to avoid the local optima trap. This strategy is characterized as BL strategy [35]. On the other hand, the worst search agent will create a large neighbourhood to increase the chances of interacting with the best ones in the case of BS strategy [35]. It will increase the quality of the bad solutions. That means two approaches will provide the advantage to the algorithm either by avoiding the local optima trap or by improving the bad solutions. Besides both will maintain diversity. When the algorithm will try to create the neighbourhood for each search agent it will do so according to the following algorithm.

**Algorithm 1: Neighbourhood Construction**

1. **function** Neighborhood_construction (hyena population)

2. Best available neighbours (List [])

    **BL** (From small to large)

    **BS** (From Large to small)

3. best_fitness_value (hyena);

4. worst_fitness_value (hyena);

5.Fitness of individual hyena population.

6.norm_fitness = (Fitness_indi_worst_fitness_value)/(best_fitness_value-

                    worst_fitness_value);

7.**return** List [norm_fitness*List.size ()];

8. **end** Neighborhood_construction

### Wellbore trajectory optimization by MOCSHOPSO

Since wellbore trajectory optimization is a non-linear optimization problem, the spotted hyena populations have been initialized semi-randomly to make the approach less chaotic. After evaluating each search agent for the wellbore trajectory optimization problem, it selects the best search agents and clusters of all obtained solutions. Then it will create a neighbourhood by using an adaptive neighbourhood framework. It will also test the fixed structure like (L5, L9, C9, C13, C21, C25) for this problem. After creating a neighbourhood according to the CA concept, they are evaluated for optimization problems along with constraint violation checking. Later they exchange information among them. If any neighbour provides a better solution than the existing population (central cell) then it will update its position otherwise the central cell will be the best solution. This process will continue until the end criterion is met. To begin, a list of the six mentioned neighbourhoods is created. This list is sorted by neighbourhood size. The concept of radius is employed in this work to quantify the size of the neighbourhood [5]. The neighbourhoods in consideration are L5, C9, C9, C13, C21, and C25. Second, whenever the algorithm requests the neighbourhood of an individual, the adaptive approach selects the neighbourhood based on the quality of the provided individual.

## Results & discussion

In this section, several statistical analyses are discussed to investigate the performance of the proposed algorithm qualitatively and quantitively for wellbore trajectory optimization. Additionally, the proposed hybrid method’s performance for trajectory optimization was compared to that of the previously utilised metaheuristic algorithm MOCPSO [10]. To validate this work, real field data (secondary) from the Gulf of Suez oil field was used [4].

### Comparative criterion

The evaluation of this work has been performed based on some statistical analysis. These parameters are the inverted generational distance (IGD), the spacing metric (SP), the maximum spread (MS), and the error ratio (ER). The next sections will describe these characteristics in detail.

#### Inverted Generational Distance (IGD)

A researcher will obtain two types of Pareto solutions based on the solution obtained. The Pareto front solution is one type, whereas the approximate Pareto front solution is another. The term IGD is used to indicate this distinction between the two. However, a true Pareto front is necessary to calculate IGD. When the true Pareto front is unavailable, non-dominated solutions serve as a baseline against which to compare. The formulation of IGD can be derived as

$$IGD(Q,Q^{*})=\frac{\sum_{i=1}^{\left|Q\right|}d(Q,Q^{*})}{\left|Q\right|}$$ (29)

Here, the difference of Euclidean distance between the true Pareto front (*Q*) and approximate (Q*) Pareto front is represented by *d*(*Q*, Q*). It is a distance-based accuracy metric. If any algorithm gives a minimum value of IGD among the comparable algorithms, then it means it has obtained a Pareto front which is very near to the true Pareto front.

#### Spacing metric (SP)

When the beginning and end of a Pareto front are unknown, it is required to determine the distribution of solutions. As such, SP is a metric that quantifies the distance variance between neighbouring vectors in the resulting non-dominated solutions. The minimum value of SP means the distance of solutions is comparatively less It indicates that solutions that are not dominated have a more balanced distribution. The formulation of the metric is as follows.


$$SP=\sqrt{\frac{1}{\left|P-1\right|}\sum_{i=1}^{\left|P\right|}{\left(d_{i}-\bar{d}\right)}^{2}}$$ (30)

Where, $d_{i}={min}_{j}(|f_{1}^{i}(\vec{x})-f_{1}^{j}(\vec{x})|+|f_{2}^{i}(\vec{x})-f_{2}^{j}(\vec{x})|)$, *i*,*j* = 1,2,…*n*, the average value of all *d*_*i*_ is represented by $\bar{d}$, and the total number of obtained Pareto solutions is represented by *P*.

#### Maximum spread (MS)

This metric is usually used to calculate the diversity of the obtained solutions and the coverage area of the solution. It is usually expressed as a difference between the two boundary solutions.


$$MS=\sqrt{\sum_{i=1}^{0}max(d(a_{i},b_{i}))}$$ (31)

Here, the maximum values in *i*^*th*^ the objective is represented by *a*_*i*_ and minimum values are represented by *b*_*i*_.

#### Error ratio (ER)

This metric defines the number of solutions that are not secured a place in the obtained Pareto optimal set. It can be mathematically expressed as follows.


$$ER=\frac{\sum_{i=1}^{n}e_{i}}{n}$$ (32)

Where, the number of vectors in the currently available non dominated set is represented by *n*, *e*_*i*_ = 0 if iϵ Pareto optimal set otherwise *e*_*i*_ = 1. But ER = 0 is an ideal case that does not mean all solutions obtained by the proposed algorithm are within Pareto optimal set.

### Comparative analysis

The statistical and analytical performances of the proposed algorithm on wellbore trajectory optimization are discussed in this section. In this analysis, the proposed algorithm is compared with the previously used algorithm MOCPSO and several other state-of-the-art algorithms such as MOSHO [21], MOCGWO [23]. Different algorithms utilized various parameters during optimization which are tabulated in Table 2. These algorithms tuned 17 variables within their constraint limit in order to produce the optimal trajectory.

**Table 2:** Parameter settings of different algorithms.

Parameter	MOCSHOPSO	MOCPSO	MOSHO	MOCGWO
**Population size**	100	100	100	100
**Archive size**	40	40	40	40
**Iterations**	100	100	100	100
**Neighbors**	10	10	-	10
**Grid size**	10	10	10	10
**Mutation**	-	0.5	-	-

**Fig 4:**
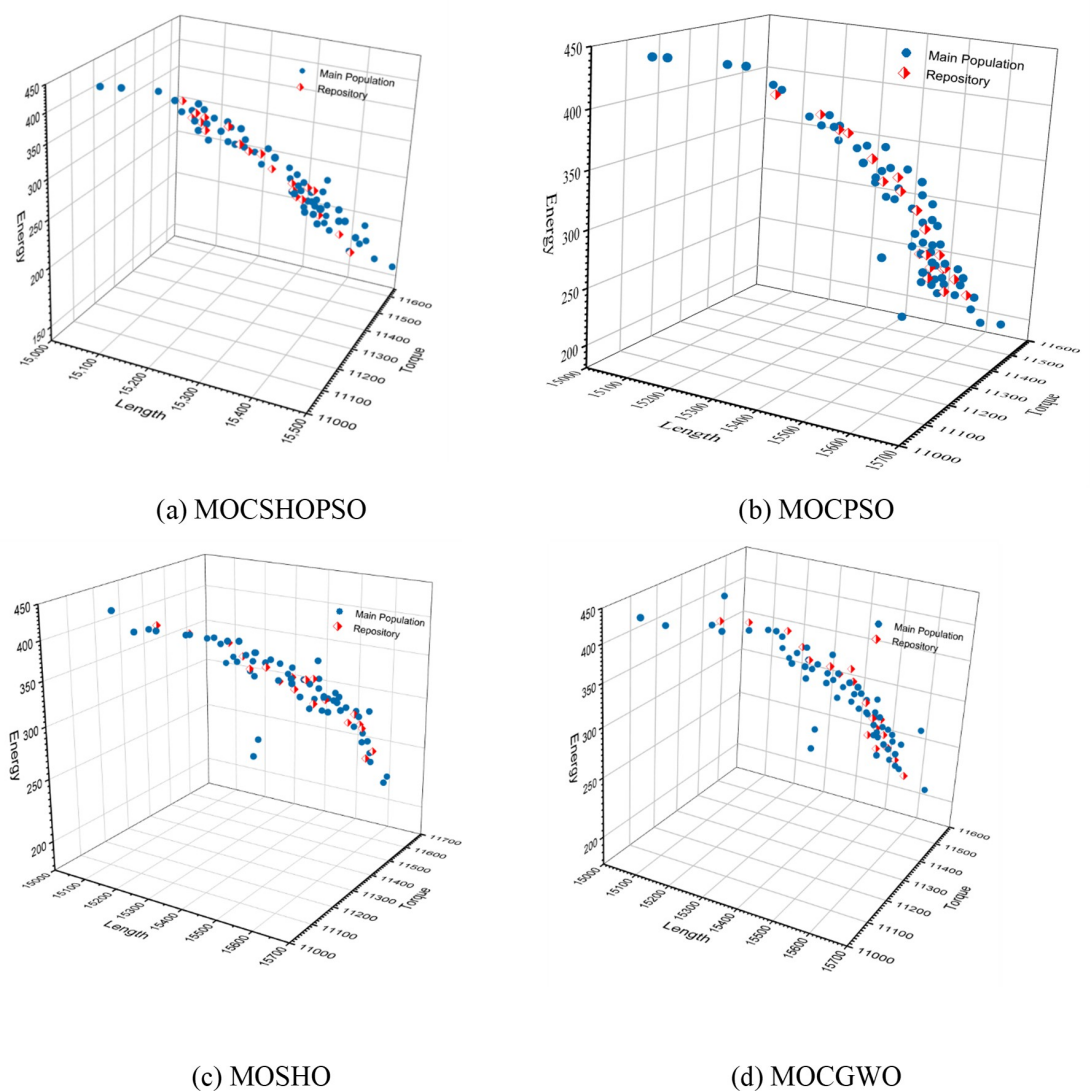
Pareto front of different algorithm. (a) MOCSHOPSO. (b) MOCPSO. (c) MOSHO. (d) MOCGWO.

Each of the algorithms under consideration obtained a Pareto front solution, as illustrated in Fig 4. As illustrated in the figure, MOCSHOPSO’s Pareto front is more convergent and distributive than those of other algorithms. The blue circle depicts the evolved search agents, while the diamond shape (red colour) reflects the archive’s non-dominated solutions. Because CA is hybridised, each search agent can communicate with its neighbours. It enhances the algorithm’s potential for local search, which ultimately enhances the algorithm’s convergence capability. Additionally, as illustrated in Fig 4, the proposed algorithm’s Pareto front has a more diverse distribution of solutions than MOSHO (Fig 4C).

**Fig 5:**
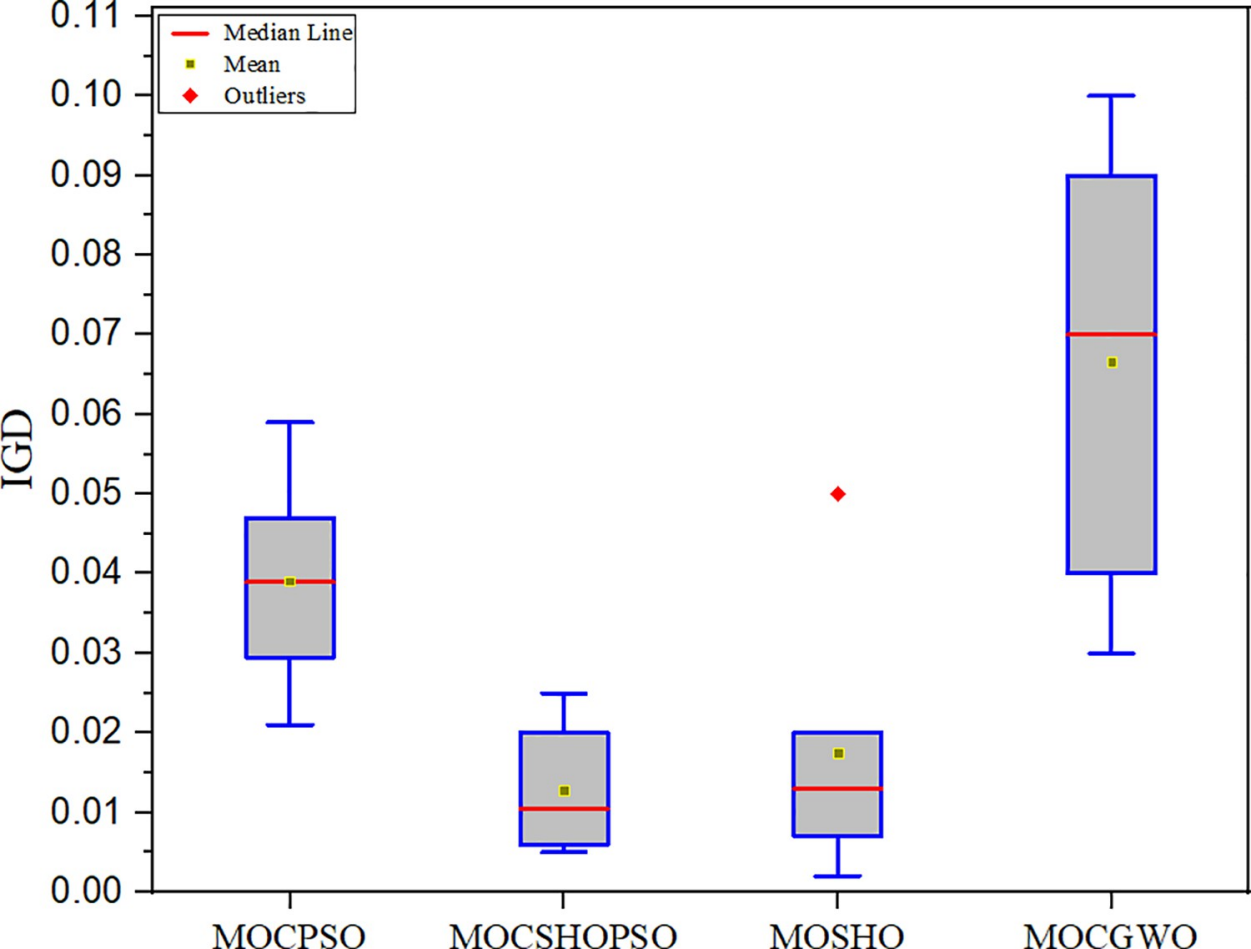
Box plot driven from the IGD comparison.

This substantial improvement is attributable to the addition of CA. By employing the characteristics of an adaptive neighbourhood, it raises the likelihood of better exploration, which ultimately aids the algorithm in providing a solution that is superior to the original. Additionally, CA’s adaptive behaviour enhanced the likelihood of improving the worst solution. However, the quality of all derived Pareto fronts is statistically assessed in this work. Each method is run 30 times during this investigation, and all box plots are generated based on the collected data. The box plot more precisely expresses the distribution of solutions than any other style of plot. Additionally, it demonstrates the data sets’ dispersion and symmetry [36]. Fig 5 illustrates the box plot for the IGD comparison metric. Whereas Table 3 contains the comparative data from the IGD analysis. The proposed approach achieved a mean value of 0.01283, which is the lowest value attained by any of the compared algorithms. Additionally, it outperformed the previously utilized MOCPSO algorithm for wellbore trajectory optimization. Furthermore, the data demonstrate that, althouth MOSHO has a larger mean value than MOCSHOPSO, but it’s obtained best solution (0.00214) is better than MOCSHOPSO. The minimum IGD value also underscored the fact that the algorithm’s Pareto front is closer to the true Pareto front than others. Due to the diffusion process of CA, it enabled the algorithm in exploring more search regions and gathering more isolated minima than was previously achievable. As a result, the gap between the obtained and true Pareto fronts has narrowed.

**Table 3:** Comparative analysis of different algorithms based on the IGD.

	MOCSHOPSO	MOCPSO	MOSHO	MOGWO
**Mean**	**0.01283**	0.03907	0.01754	0.06667
**Variance**	0.00009	0.00015	0.00018	0.00098
**Std**	0.00948	0.01224	0.01341	0.03130
**Best**	0.00501	0.02109	0.00214	0.03001
**Worst**	0.02502	0.05903	0.05103	0.10680

**Fig 6:**
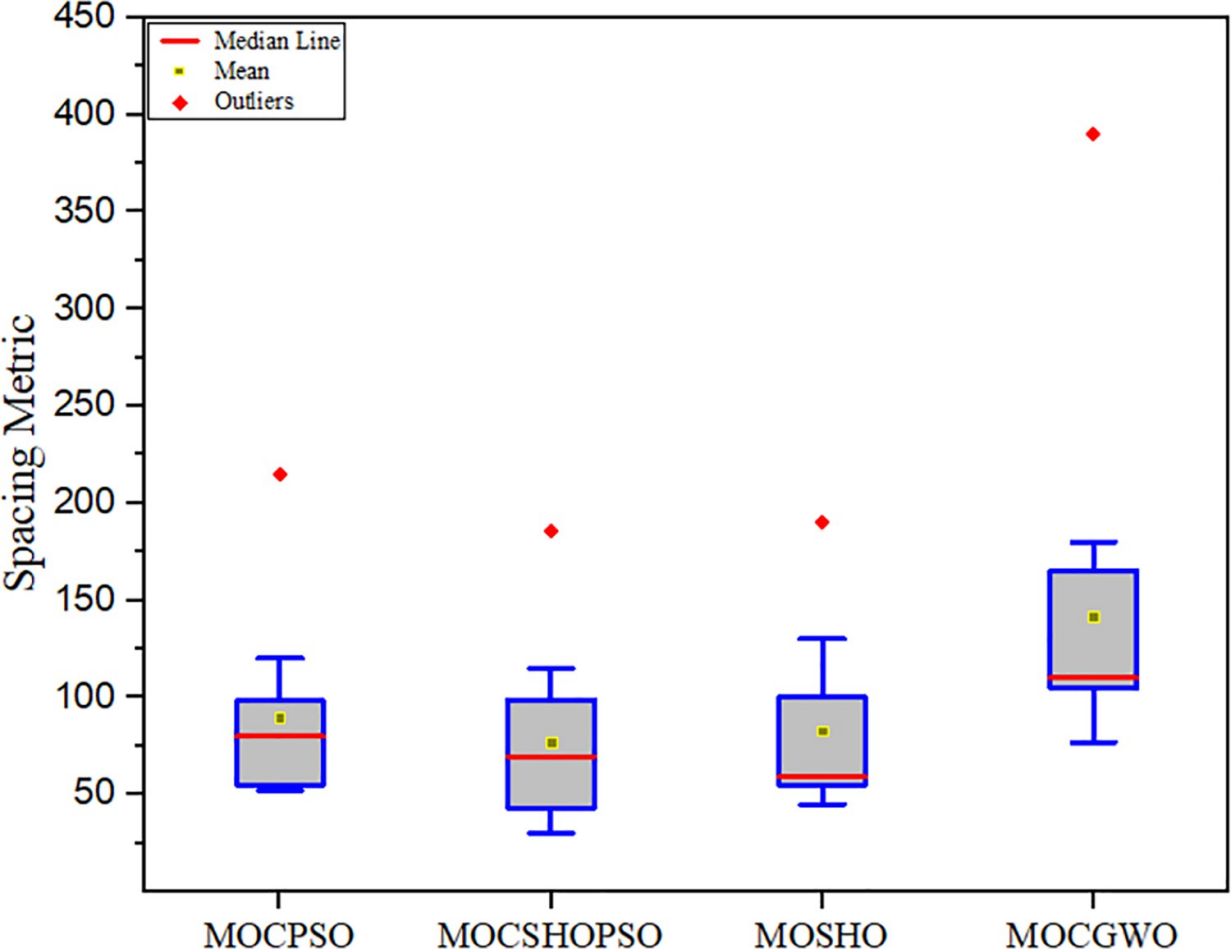
Box plot, driven from the spacing metric comparison.

Table 4 summarises the SP analysis’s findings. The proposed technique achieved the lowest mean value among comparable algorithms (77.0527). This shows that the solutions generated by this algorithm are very evenly spaced. Throughout the hunting process, the recommended algorithm’s search agents used PSO’s velocity update feature to update their positions. This aided the algorithm in producing this positive outcome. The decrease in the value of $\vec{h}$ and $\vec{F}$ has also played a role in this problem. Fig 6 illustrates the box plot of the spacing metric analysis.

**Table 4:** Comparative analysis of different algorithms based on the spacing metric.

	MOCSHOPSO Method	MOCPSO	MOSHO	MOGWO
**Mean**	**77.0527**	89.7273	82.0903	141.8186
**Variance**	1798.3758	3158.4003	679.1762	4286.2398
**Std**	42.4072	56.1996	26.0610	65.4693
**Best**	30.1735	51.7924	50.3976	77.1458
**Worst**	186.7689	215.4687	190.7658	390.3698

**Fig 7:**
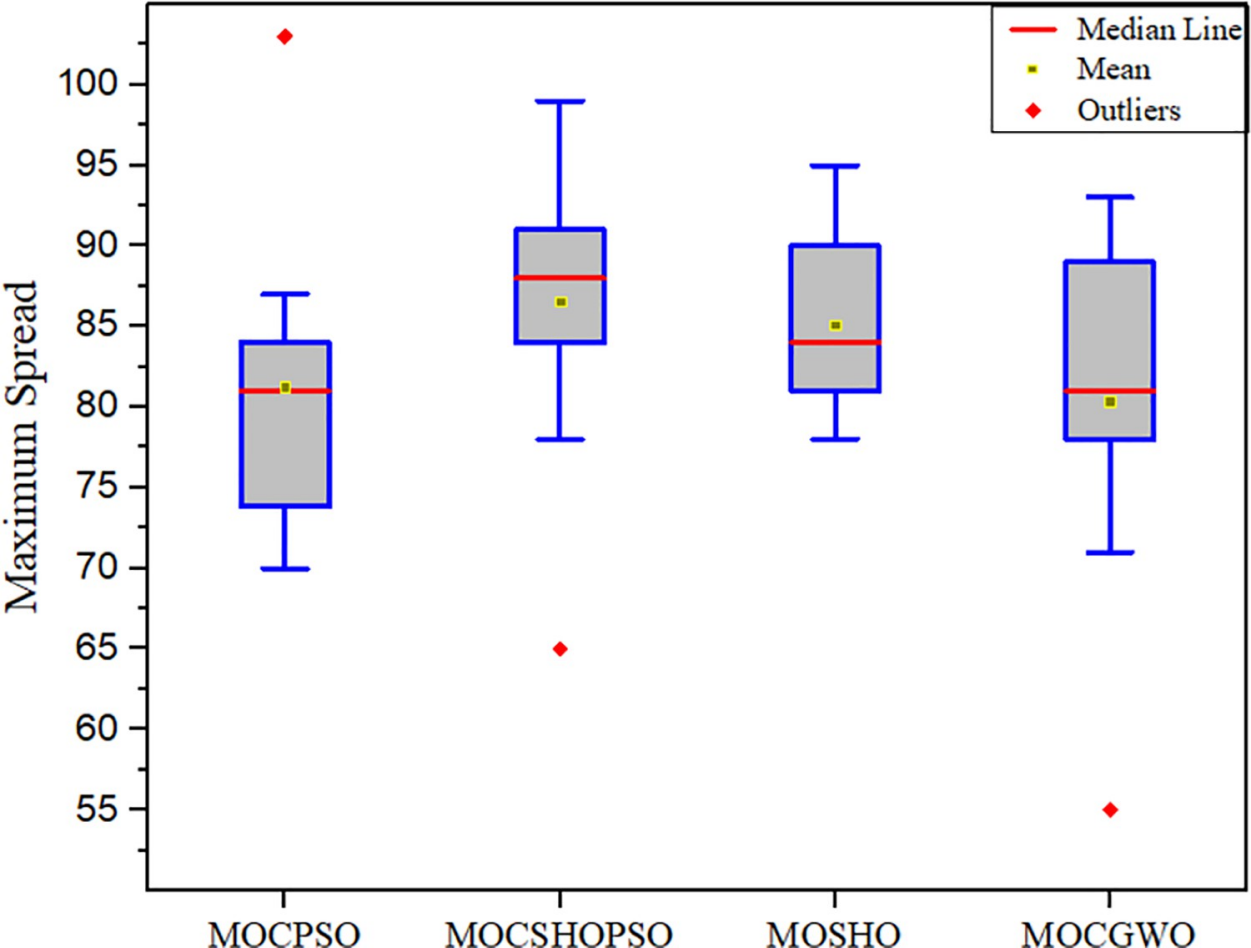
Box plot, driven from the maximum spread comparison.

**Fig 8:**
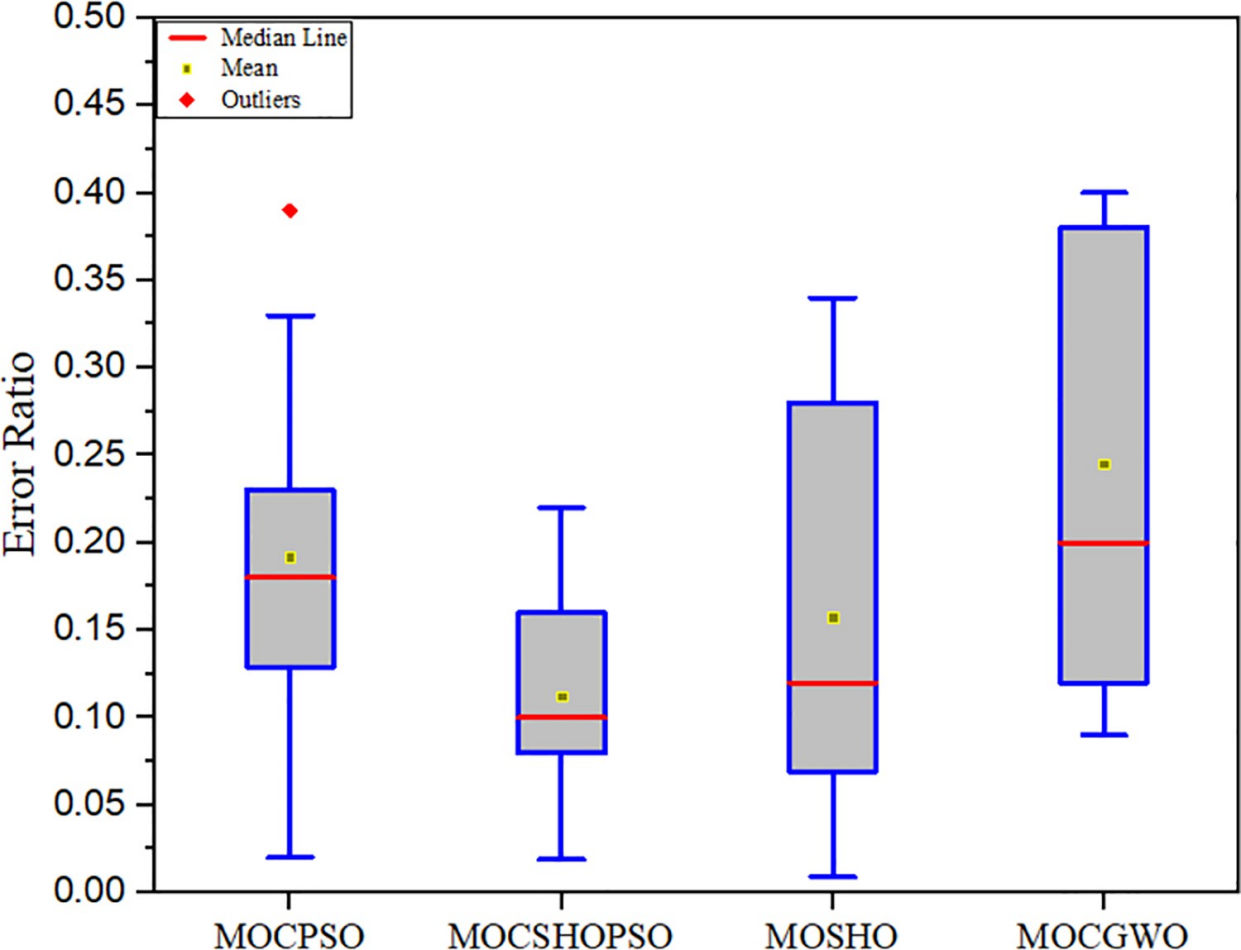
Box plot, driven from the error ratio comparison.

The proposed technique is also analysed in terms of MS and ER metrics in this work. The MS and ER experimental results are summarized in Tables 5 and 6, respectively. MS and ER box plots are presented in Figs 7 and 8, respectively.

**Table 5:** Comparative analysis of different algorithms based on the maximum spread.

	MOCSHOPSO	MOCPSO	MOSHO	MOGWO
**Mean**	**86.5454**	81.2554	85.0909	80.3636
**Variance**	25.7542	38.0192	28.3702	39.4327
**Std**	5.0748	6.1659	5.3263	6.2795
**Best**	65.0012	70.0001	78.1003	55.0312
**Worst**	99.0104	103.3004	95.1754	91.3245

**Table 6:** Comparative analysis of different algorithms based on the error ratio.

	MOCSHOPSO	MOCPSO	MOSHO	MOGWO
**Mean**	**0.1122**	0.1921	0.1575	0.2454
**Variance**	0.0012	0.0021	0.0101	0.0235
**Std**	0.0346	0.0458	0.1004	0.1532
**Best**	0.0191	0.0213	0.0091	0.0901
**Worst**	0.2201	0.3912	0.3410	0.3912

As indicated by the experimental findings, MOCSHOPSO has the highest spread and the lowest error ratio among the comparison algorithms. The proposed algorithm achieves a mean value of 86.5454 for the MS, which is much greater than the value obtained by existing algorithms. This is due to the enhancement of MOSHO’s exploration capability. Additionally, it was noted that the proposed algorithm more uniformly distributes non-dominated solutions than others. The proposed method results in a mean of 0.1122 for ER. This suggests in certain search regions, it has a lower proportion of significant non-dominated solutions than in others. Additionally, it has been demonstrated that MOCSHOPSO is capable of collecting more isolated significant minima than other algorithms. This favourable finding also demonstrates the critical nature of including the slow diffusion mechanism of CA. Because an algorithm can achieve the lowest ER value only when the number of missing non-dominated solutions is minimized.

**Fig 9:**
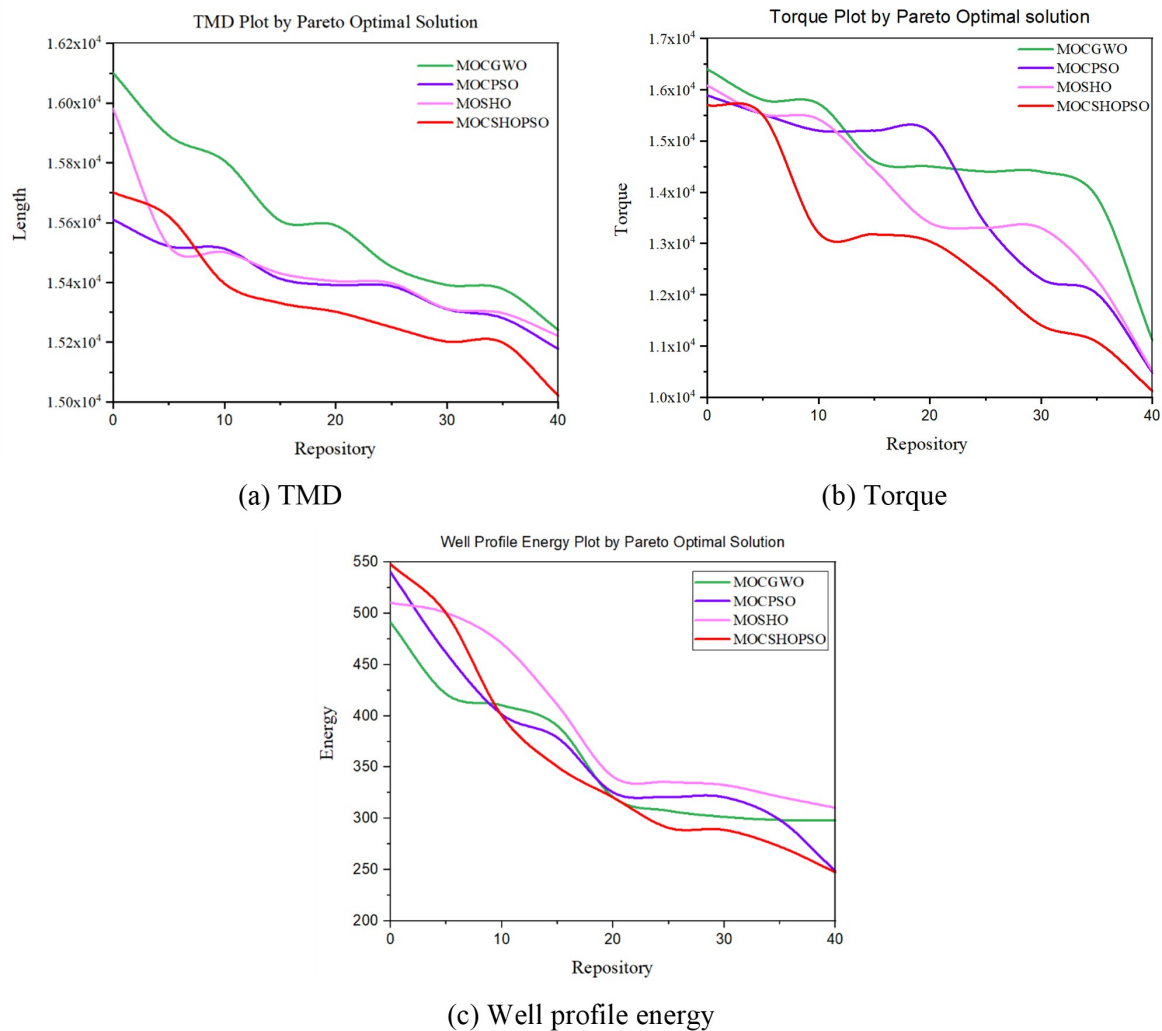
Repository solutions for the studied three objectives. (a) TMD. (b) Torque. (c) Well profile energy.

However, the proposed method has been compared qualitatively. Fig 9 depicts the data from each repository in terms of three objective functions. Before plotting, the values in the repository are sorted. MOCSHOPSO surpassed all other algorithms in terms of TMD, as shown in Fig 9(A). Though MOCPSO began with a lower value, MOCSHOPSO eventually reached the optimal minima faster.

From a similar analysis of torque, it is clear that the torque generated by MOCSHPSO is significantly less than that calculated by other methods. MOSHO performs similarly to the previously used algorithm MOCPSO in this case, while it performs better in repositories 10 and 20. Additionally, the suggested approach outperforms the comparative algorithm in terms of well profile energy. This suggests that the design parameter obtained will result in a less complex and cost-effective trajectory design.

**Fig 10:**
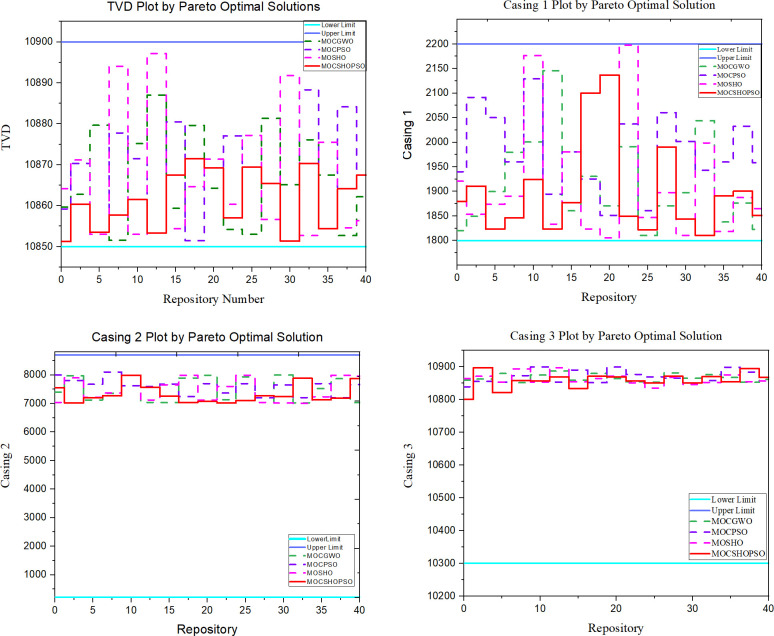
Comparison plot for operational constraints. (a) TVD. (b) Casing 1. (c) Casing 2. (d) Casing 3.

Throughout the optimization process, the algorithms have taken into account the geological and operational constraints associated with wellbore trajectory optimization, as shown in Table 1. The algorithmic management of constraints and their required boundaries is depicted in Fig 10. It is shown that MOCSHPSO managed constraints effectively and with very little fluctuation. This is because the initialization is semi-random rather than random. It has resulted in less chaotic handling of constraints.

#### The performance of MOCSHOPSO with different neighbourhood structures

**Fig 11:**
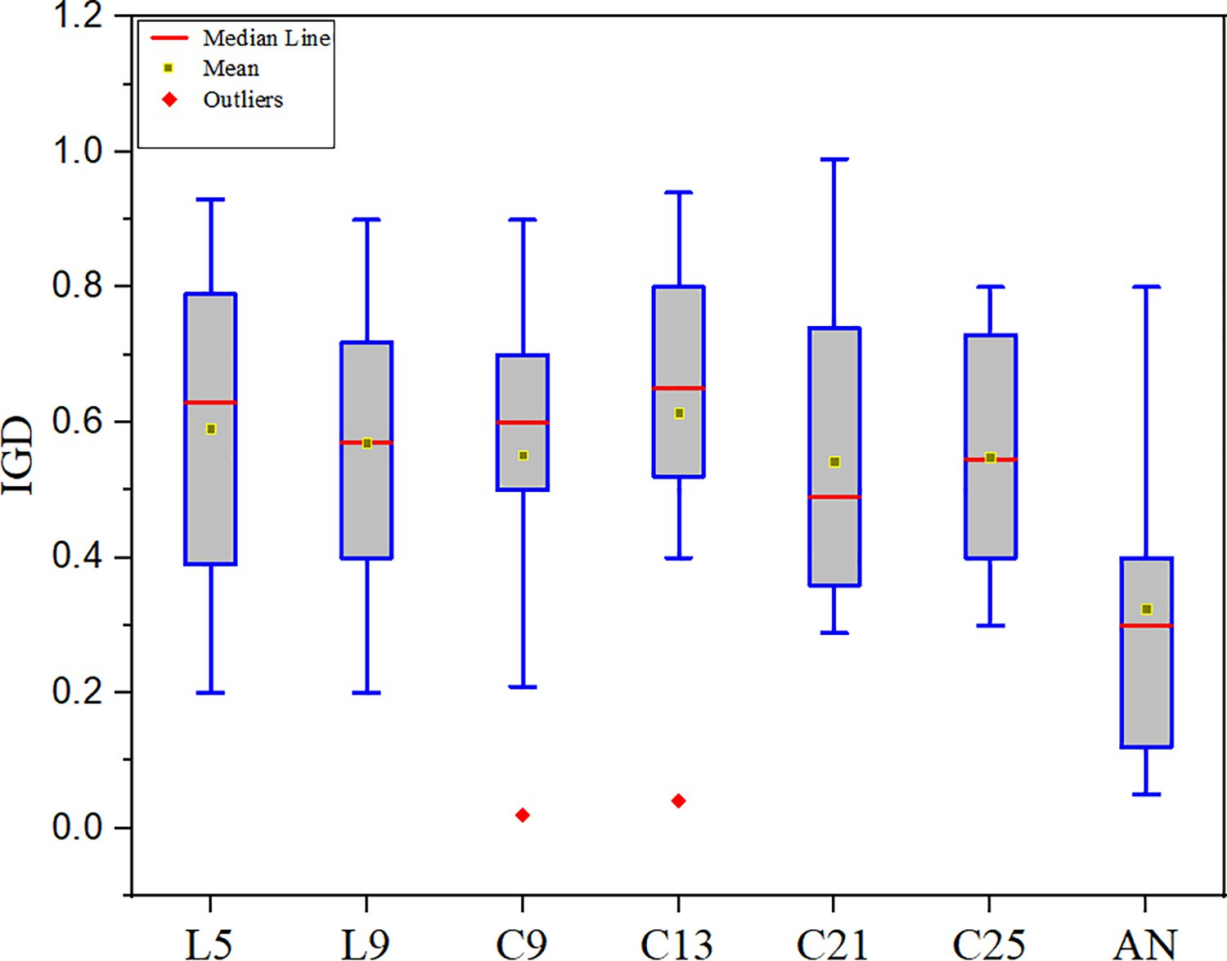
Box plot of IGD metric for different neighbourhood.

Six distinct variations of MOCSHOPSO are described in this section, each based on a different neighbourhood topology in addition to the adaptive one. These versions were evaluated against one another in terms of performance and statistical analysis. However, for wellbore trajectory optimization, MOCSHOPSO with C21 performed marginally better than its other versions in the case of fixed topology. As a result, C21 is recognized as a suitable neighbourhood structure for forming the optimal MOCSHOPSO. However, when adaptable neighbourhoods are used, they slightly outperform all fixed neighbourhood topologies. Table 7 summarizes the IGD metric analysis for each neighbourhood topology, and Fig 11 illustrates the box plot. According to Table 7, the mean value for adaptive neighbourhoods is 0.3251 which is much less than the mean value for fixed neighbourhoods. C21 has a mean value of 0.5975 among the fixed topologies. As illustrated in Fig 11, adaptive neighbourhood (AN) has better distribution than others. Additionally, the Friedman test was used to determine the optimal neighbourhood topology among the neighbourhood topologies. It is a frequently used statistical test for multiple comparisons that is inherently non-parametric [37]. Table 8 has tabulated the data for the Friedman test analysis.

**Table 7:** IGD experimental result for different neighbourhood.

	L5	L9	C9	C13	C21	C25	AN
**Mean**	0.5921	0.5689	0.5521	0.6155	**0.5431**	0.5484	**0.3251**
**Variance**	0.0265	0.0321	0.0301	0.0421	**0.0264**	0.0386	**0.0103**

**Table 8:** Friedman test result for individual ranking and P-value.

Neighbourhood Functions	Rank	P value
L5	3.6012	0.0512
L9	2.9145	
C9	3.4365	
C13	3.6191	
C21	2.8906	
C25	3.3654	
AN (Adaptive Neighborhood)	2.4512	

According to the Friedman test data, it can be concluded that AN performed better than other topologies. This is the optimal neighbourhood topology for optimizing the wellbore trajectory.

Radius also plays an important role during neighbourhood construction. If the radius becomes too small then it may fall into the local optima trap and may miss some significant minima. On the other hand, if it becomes too large it gives more attention to global search which may lead to slow convergence.

**Fig 12:**
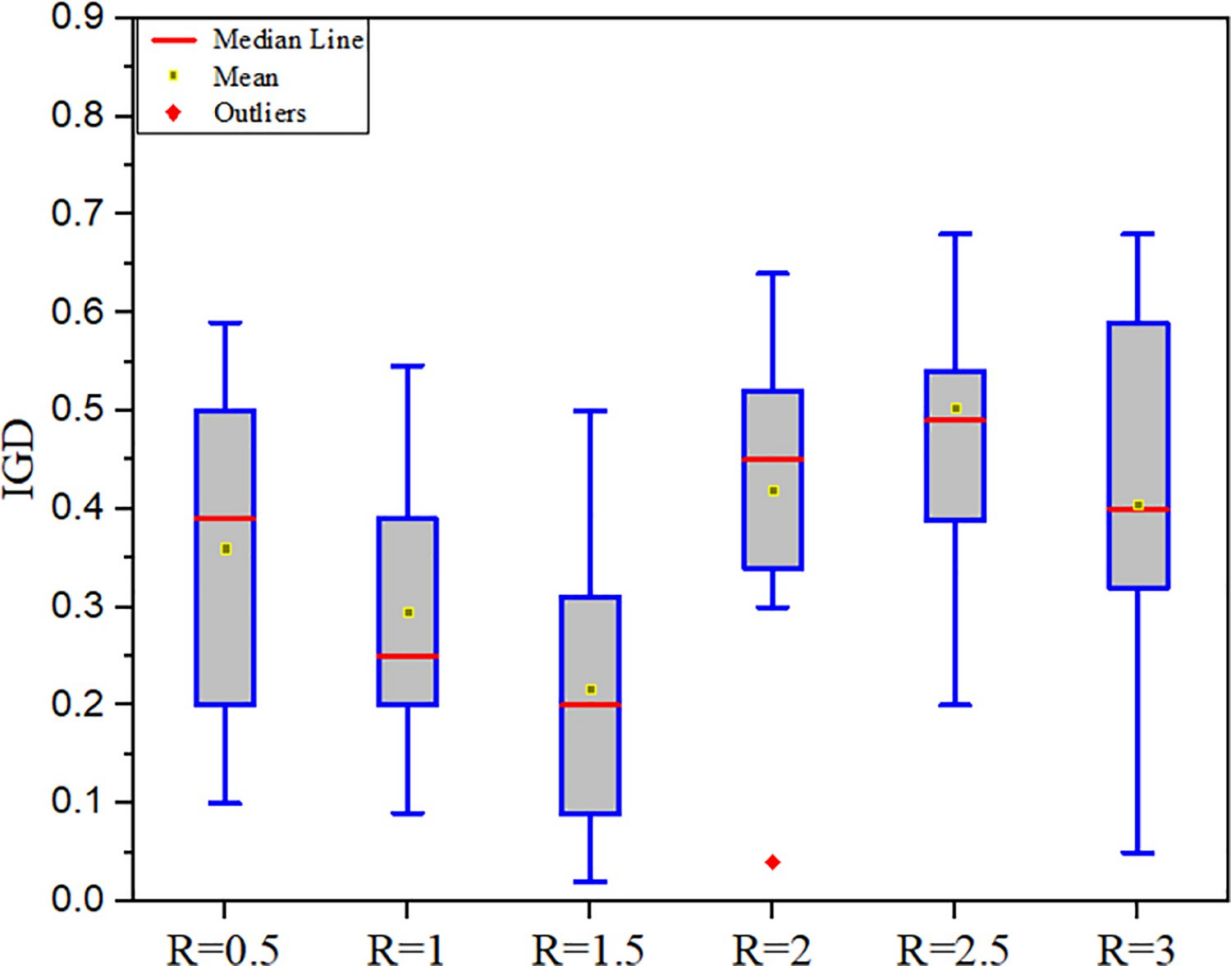
IGD analysis with different values of radius.

In this paper, various radius values are evaluated to determine the optimal radius for the neighbourhood topology. Table 9 contains the experimental data for IGD analysis, and Fig 12 depicts the box plot. According to the experimental results, the optimal radius for neighbourhood formation is R = 1.5. It has a mean of 0.2166, which is the smallest number among the compared radii.

**Table 9:** Experimental data of IGD metric for comparative radius.

	R = 0.5	R = 1	R = 1.5	R = 2	R = 2.5	R = 3
**Mean**	0.3698	0.2951	**0.2166**	0.4231	0.5081	0.4051
**Variance**	0.0177	0.0201	**0.0164**	0.0241	0.0512	0.0401

#### Sensitivity analysis

It is a qualitative analytical technique used to ascertain the significance of tuning parameters utilized throughout the optimization process. Different tuning parameters contributed identically to attaining the minimal value for three optimization objectives throughout the optimization. There are seventeen tuning variables in this work, each with its constraints. This study will determine which parameter contributed the most and which one contributed the least. Among the two types of sensitivity analysis, local sensitivity analysis expresses the relevance of each variable but does not reveal any correlation information in the case of multi-objective optimization. However, with the global sensitivity analysis, adjustment of any variable, a correlation between all the objectives is recognized. Due to the nonlinear nature of wellbore trajectory optimization, global sensitivity analysis was performed in this case. In this paper, the Spearman correlation coefficient analysis was used to determine the global sensitivity [24]. Table 10 summarizes all experimental data. If a variable achieves the highest value among the 17 variables, it contributes the most during optimization. Additionally, this indicates that the optimization is more dependent on the variable than on the others. The three highest values have been highlighted in Table 10 to indicate the three most significant variables for each objective.

**Table 10:** Spearman correlation coefficient analysis for the decision parameters.

Symbol	MOCSHOPSO			MOCPSO			MOSHO			MOCGWO		
	TMD	Torque	Energy	TMD	Torque	Energy	TMD	Torque	Energy	TMD	Torque	Energy
**∅**_**1**_	**0.942**	**0.796**	0.014	0.813	0.972	-0.917	0.632	0.739	-0.894	0.307	0.976	0.071
**∅**_**2**_	**0.842**	**0.189**	**0.798**	-0.214	-0.442	0.623	-0.058	-0.278	0.379	0.809	0.493	0.111
**∅**_**3**_	-0.346	-0.215	**0.762**	0.3079	-0.1818	0.3338	0.354	-0.243	0.287	0.401	-0.801	0.226
***θ***_**1**_	-0.023	-0.257	0.036	-0.049	-0.041	0.076	0.213	0.254	-0.025	-0.637	0.098	-0.068
***θ***_**2**_	0.054	-0.201	-0.054	-0.066	0.060	-0.176	-0.315	-0.504	0.521	-0.378	-0.065	-0.435
***θ***_**3**_	**0.543**	0.041	-0.394	-0.149	-0.280	0.207	0.057	0.013	0.186	-0.301	-0.051	-0.073
***θ***_**4**_	-0.365	-0.325	-0.114	-0.123	-0.103	-0.052	-0.269	-0.166	0.257	-0.003	-0.047	-0.732
***θ***_**5**_	-0.054	0.029	0.023	-0.030	0.266	-0.403	-0.369	0.265	-0.301	0.376	0.317	-0.953
***θ***_**6**_	0.052	0.075	0.029	-0.019	-0.039	0.106	-0.205	-0.096	0.326	-0.356	-0.193	-0.054
***D***_***kop***_	-0.135	0.0265	-0.154	-0.012	0.081	-0.244	-0.165	-0.436	0.396	-0.341	-0.019	-0.079
***D***_***D***_	-0.156	-0.092	-0.198	-0.599	-0.480	0.324	-0.526	-0.069	0.136	0.146	-0.009	0.298
***D***_***B***_	-0.057	0.016	-0.079	-0.269	-0.002	-0.148	-0.458	0.245	-0.186	-0.175	0.058	-0.345
***T***_**1**_	0.456	**0.684**	-0.186	0.587	0.800	-0.864	0.547	0.867	-0.753	0.245	0.634	0.178
***T***_**2**_	-0.079	-0.096	-0.045	-0.241	-0.343	0.463	-0.368	-0.125	0.006	0.186	-0.089	0.076
***T***_**3**_	-0.403	-0.301	0.301	-0.003	0.063	-0.459	0.076	0.005	0.069	0.247	-0.086	-0.345
***T***_**4**_	0.126	0.138	**0.187**	0.171	0.041	0.113	0.056	0.338	-0.856	-0.365	-0.326	-0.289
***T***_**5**_	-0.886	-0.160	-0.768	-0.246	0.094	-0.267	-0.075	0.097	-0.325	-0.086	-0.396	-0.875

## Conclusion

In this work, a noble hybrid algorithm named, MOCSHOPSO has been proposed and demonstrated for wellbore trajectory optimization considering the TMD, well profile energy and torque. The hybridization is done with the incorporation of cellular automata (CA) and particle swarm optimization (PSO). The incorporation of CA has significantly improved the exploration capability of the algorithm. Besides the velocity update mechanism of PSO significantly contributed to the hunting mechanism of MOCSHOPSO. The performance of the proposed algorithm in wellbore trajectory optimization is compared with the MOCPSO, MOSHO, and MOCGWO. This algorithm showed superior performances than all the three established algorithms. The significant improvements of the original algorithm have been validated through different statistical analyses. It has achieved the lowest IGD, SP and ER value on the other hand it has also given maximum value for MS. The hybridization has also significantly improved the capability of tracing the isolated minima which are validated by the lower value of ER. Moreover, an adaptive neighbourhood mechanism has proposed which has shown better performance than the fixed neighbourhood topologies. Additionally, the proposed algorithm has shown significant improvements during geophysical and operational constraints handling of the wellbore trajectory optimization. Semi-random initialization of the search agents has helped the algorithm to achieve this less chaotic constraint handling. Moreover, this algorithm has provided optimum parameters for trajectory design which will help the industry to design a cost-effective and less complex trajectory design.

## Supporting information

S1 Appendix Table A1. Description of the variables.
(DOCX)

## Abbreviations

ABC: Artificial bee Colony Algorithm
ACO: Ant Colony Optimization
AN: Adaptive Neighbourhood
BA: Bat algorithm
BFO: Bat Flight optimization
CA: Cellular Automata
CSO: Crow Search Algorithm
DS: Differential Search
ER: Error Ratio
GA: Genetic Algorithm
GWO: Grey Wolf Optimization
HS: Harmony Search
HCSO: Hybrid Cuckoo Search Optimization
IGD: Inverted Generational Distance
KOP: Kick of Point
MCM: Minimum Curvature Method
MO: Multi-Objective
MOCPSO: Multi-Objective Cellular Particle Swarm Optimization
MOCGWOPSO: Multi-Objective Cellular Grey Wolf Optimization with Particle Swarm Optimization
MOGWO: Multi-Objective Grey Wolf Optimization
MS: Maximum Spread
PSO: Particle Swarm Optimization
RCM: Radius Curvature Method
SHO: Spotted Hyena Optimizer
SP: Spacing Metric
TMD: True Measured Depth
TVD: True Vertical Depth
