# Supplementary material for: A hybrid multi objective cellular spotted hyena optimizer for wellbore trajectory optimization
Source: PLoS One. 2022 Jan 27;17(1):e0261427. doi: 10.1371/journal.pone.0261427 (PMC8794190; doi:10.1371/journal.pone.0261427)
Supplement: S1 Appendix — Description of the variables. (DOCX) [file pone.0261427.s001.docx]

**S1 Appendix**

**Table A1.** Description of the variables.

| **Symbol** | **Name of the variables** |
| --- | --- |
| $\emptyset_{1}$ | First hold angle in degree |
| $\emptyset_{2}$ | Second hold angle in degree |
| $\emptyset_{3}$ | Second hold angle in degree |
| $\theta_{1}$ | Azimuth angle at kick of point in degree |
| $\theta_{2}$ | Azimuth angle at end of the first build portion in degree |
| $\theta_{3}$ | Azimuth angle at end of first hold section in degree |
| $\theta_{4}$ | Azimuth angle at end of second drop portion in degree |
| $\theta_{5}$ | Azimuth angle at the end of second hold section in degree |
| $\theta_{6}$ | Azimuth angle at the end of third build portion in degree |
| $T_{1}$ | Dogleg severity of first build portion. ^0^/100 feet |
| $T_{2}$ | Dogleg severity of first hold section. ^0^/100 feet |
| $T_{3}$ | Dogleg severity of second drop portion. ^0^/100 feet |
| $T_{4}$ | Dogleg severity of second hold portion. ^0^/100 feet |
| $T_{5}$ | Dogleg severity of third build portion. ^0^/100 feet |
| TVD | True vertical depth of the well at total depth (TD) in feet |
| $D_{kop}$ | True vertical depth of the kickoff point, feet |
| $D_{D}$ | True vertical depth of the well at the top of drop off section, feet |
| $D_{B}$ | True vertical depth of the well at the end of drop off section, feet |
| HD | Lateral or horizontal length (HD), feet |
| TMD | True measure depth in feet |
